# St. Louis Odontological Society

**Published:** 1866-07

**Authors:** G. Granville Samuel

**Affiliations:** St. Louis Odontological Society


### ST. LOUIS ODONTOLOGICAL SOCIETY.

On the 26th day of Octobor 1865, a number of resident dentists of the city of St. Louis assembled and formed themselves into a society. A constitution and by-laws being adopted, the following officers were elected for the ensuing year:—

President, E. Hale, Jr. Vice President, J. Payne, Corresponding Secretary, G. G. Samuel, Recording Secretary, G. H. Silvers, Executive Committee, A. D. Sloan, C. W. Gill, J. Payne.

Knowing it to be indispensibly necessary that the dentist should possess a thorough and practical knowledge of the anatomical structure of the human system, as well as the practical functions of the various organs of the same, it was unanimously resolved, to immediately enter upon a course of anatomical studies, and to employ a competant person to deliver a course of such lectures, and to demonstrate the same in the desecting room.

Whereupon the services of the well-known popular and scientific lecturer and anatomist, Dr. John J. McDowell of this city were secured, and he immediately entered upon the duties assigned him, and he in a very able and satisfactory manner discharged said duties, having dissected two entire subjects during his course of study and instructions.

Stimulated by the manifest advantages derived from this course of study and instructions, the society are determined to continue on in the good work and prosecute their labors until they shall have acquired practical and scientific knowledge of the anatomy, physiology and pathalogy of the human system, and the cloud which has heretofore darkened and obscured many of the avenues to success in their professional practice, shall have disappeared before the light of science, when each member of the Odontological Society may be weighed in the balance, and not found wanting, but stand in the scale of respectability and usefulness on a level with the now more honored and learned Medical Profession.

Giving indubitable evidence that the St. Louis Odontological Society is founded, not in things perishable, but on the broad and immutible *fiat* of Nature.

G. Granville Samuel, Cor. Sec'y.
St. Louis Odontological Society.

